# Treatment of Elderly Patients with Acute Symptomatic OVCF: A Study of Comparison of Conservative Treatment and Percutaneous Kyphoplasty

**DOI:** 10.3389/fsurg.2022.942195

**Published:** 2022-07-13

**Authors:** Dejun Yu, Zuyao Liu, Hongqing Wang, Ran Yao, Fu Li, Yang Yang, Fenglong Sun

**Affiliations:** ^1^ Second Department of Orthopedics, Beijing Rehabilitation Hospital, Capital University of Medical Sciences, Beijing, China; ^2^ Department of Orthopaedics, Beijing Rehabilitation Hospital, Capital University of Medical Sciences, Beijing, China

**Keywords:** OVCF, percutaneous kyphoplasty, pain, comparison of conservative, elderly patients

## Abstract

**Objective:**

The present study was designed for the contrastive analysis of conservative and percutaneous kyphoplasty (PKP) on pain severity and recovery of injured vertebrae in elderly patients with acute symptomatic osteoporotic vertebral compression fracture (OVCF).

**Methods:**

A total of 60 elderly patients with acute symptomatic OVCF were divided into two groups according to different treatment protocols, with 30 patients in each group. Patients in the Con group received conservative treatment, while patients in the PKP group received percutaneous kyphoplasty treatment. Clinical evaluation included the visual analogue scale (VAS), the Dallas pain questionnaire, the vertebral body leading edge height, the Cobb angle of injured vertebrae, the MOS item short-form health survey (SF-36), the Barthel index, and the mini-mental state examination (MMSE).

**Results:**

At 3 days, 3 months, and 6 months post-treatment, the score of VAS and the Cobb angle of injured vertebrae in patients of the PKP group were all significantly lower than those in the Con group (*P *< 0.05), while the height of vertebral body leading edge in patients of the PKP group was significantly longer than that in the Con group (*P *< 0.05). At 6 months post-treatment, the scores of the four dimensions of the Dallas pain questionnaire scale in the PKP group were all significantly lower than those in the Con group (*P *< 0.05), while the score of SF-36 (PCS), SF-36 (MCS), and Barthel index in patients of the PKP group were all significantly lower than those in the Con group (*P *< 0.05), and there was no significant difference in the scores of MMSE between these two groups (*P *> 0.05).

**Conclusion:**

Compared with conservative treatment, PKP treatment of elderly patients with acute symptomatic OVCF provides rapid pain relief, restoration of damaged vertebral body height, correction of Cobb's angle, and improved quality of life.

## Introduction

Osteoporosis is a systemic bone disease in which bone density and bone quality decrease due to various reasons, and the microstructure of bone is destroyed, resulting in increased bone fragility, which is prone to fractures (1, 2). Epidemiological data show that the incidence of osteoporosis in the population over 60 years old in China is about 36%, and the incidence in women is slightly higher than that in men (3, 4). Due to huge population in China, there are about 100 million osteoporosis patients. Fracture is the most common complication of osteoporosis, among which vertebral body compression fracture is the most common, namely, osteoporotic vertebral compression fracture (OVCF) (5). Severe pain and mobility dysfunction are the main clinical manifestations of OVCF patients (6). Therefore, the purpose of OVCF therapy is to relieve the pain symptoms, improve the activity ability of patients, and restore their self-care ability (7).

Currently, there is still no absolutely uniform standard for treating OVCF, and the most widely accepted and clinically implemented methods are mainly minimally invasive surgical schemes and conservative methods (8). The conservative treatment protocol for OVCF is relatively uniform, that is, bed rest, external fixation support, analgesic drug treatment, physical therapy, etc. The disadvantages of conservative treatment of OVCF are obvious, including slow pain relief, long treatment time, and the long-term bed rest easily causes complications such as bedsores, respiratory and urinary tract infections, and constipation (9). Percutaneous kyphoplasty (PKP) is one well-known percutaneous procedure effective in relieving pain caused by acute and subacute vertebral compression fracture (10). However, although OVCF is the most common indication for PKP, provides rapid pain relief, and has an acceptable safety profile when used by skilled physicians, there are still risks of surgery and refractures in elderly patients with OVCF (11). Therefore, the comparative study of the benefits of OVCF patients in different treatment modalities is of great significance to the clinical development of OVCF treatment protocols. In the present study, we compared the effects of conservative and PKP therapy on the recovery of injured vertebral bodies and pain in OVCF patients.

## Data and Methods

### Selection Criteria

A total of 60 elderly patients with acute symptomatic OVCF were recruited for the present study in Beijing Rehabilitation Hospital from January 2020 to December 2020. Also, all patients were informed about the content of this study and signed informed consent. Beijing Rehabilitation Hospital Ethics Committee is responsible for the ethical review and supervision of this study.

Inclusion criteria are as follows: (1) fracture time lower than 2 weeks, (2) radiographically confirmed osteoporotic vertebral compression fracture, (3) age >60 years, (4) significant back pain but no symptoms of nerve damage, and (5) osteoporosis confirmed by bone densitometry.

Exclusion criteria are as follows: (1) patients with communication disorders, mental disorders, intellectual disabilities, and other reasons who cannot complete the subjective assessment, (2) patients with spinal or skin infections, (3) patients with coagulation disorders, malignant tumors, limb fractures, bone metabolic diseases, or other tissue and organ dysfunction, (4) patients with drug, alcohol, or other drug addiction, (5) incomplete baseline data, and (6) inability to complete a 6-month follow-up after initial treatment.

### Treatment Protocol

Patients in the Con group received conservative treatment as follows: rest in bed to reset the fractured vertebral body, exercise the function of the lumbar back muscles, wear a spinal brace to get out of bed for exercise, and walk under the protection of the waist circumference according to the actual situation of the patient. At the same time, antiosteoporosis treatment and drug analgesic treatment were given.

Patients in the PKP group received percutaneous kyphoplasty treatment as follows: pedicle approach, X-ray localization, and local anesthesia. The pedicle was entered along the puncture point under fluoroscopy, the balloon was located at the anterior third-fourth of the vertebral body, the contrast agent was injected under continuous fluoroscopic monitoring, the balloon was slowly expanded, the balloon pressure was observed and the pressure was stopped when appropriate, the contrast agent was withdrawn, and the balloon was withdrawn. At last, the appropriate amount of bone cement was dropped into the vertebral body under fluoroscopic monitoring.

### Data Collection and Clinical Evaluation

(1) The baseline data of patients in this study, including gender, age, body mass index (BMI), fracture time, fractured segment, and hospital stay time, were collected.
(2) The visual analogue scale (VAS) scores of the two groups before treatment, 3 days after treatment, 3 months after treatment, and 6 months after treatment were compared. The full score of the scale was 10. A higher score indicated more severe pain (11).
(3) The Dallas pain questionnaire (DPQ) scores before treatment, 3 days after treatment, 3 months after treatment, and 6 months after treatment were compared between the two groups. DPQ included four aspects of daily activity (da), work and entertainment (wl), anxiety and depression (ad), and social interest (SI). A higher score indicated more severe pain (12).
(4) The MOS short-term (SF-36) scores of the health survey before treatment, 3 days after treatment, 3 months after treatment, and 6 months after treatment were compared between the two groups. SF-36 contained 36 questions, covering eight dimensions, including body function, body role, body pain, general health, vitality, social function, emotional role, and mental health, with a maximum score of 100 points in each dimension. The higher the score in each dimension, the better the state (13).
(5) The scores of the Barthel index (0–20 points) of the two groups before treatment, 3 days after treatment, 3 months after treatment, and 6 months after treatment were compared. The scale included 10 items for evaluating an individual’s daily functions, including diet, bathing, appearance, clothing, defecation, urination, self-use of the toilet, transportation ability, activity ability, and going upstairs and downstairs (14). The full score was 100. The higher the score, the stronger the patient's daily living ability.
(6) The mini-mental state examination (MMSE) scores before treatment, 3 days after treatment, 3 months after treatment, and 6 months after treatment in the two groups were compared. MMSE includes six aspects including direction, recording, attention, calculation ability, memory, and language ability, and the score <27 indicated the existence of cognitive dysfunction (15).

### Radiographic Evaluation

All patients underwent standing anteroposterior and lateral X-rays before treatment and at 3 days, 3 months, and 6 months post-treatment to determine the vertebral body leading edge height and the Cobb angle of injured vertebrae.

### Statistical Analysis

SPSS19.0 software was used for statistical analysis in the present study. Chi-square tests were used to compare the difference between categorical variables. The Kolmogorov–Smirnov test was used to check whether quantitative data conformed to a normal distribution, and data that conformed to a normal distribution were presented as mean ± standard deviation; and unpaired Student's *t*-test was used to compare differences and calculate *P*-values. *P*-values less than 0.05 indicated significant differences.

## Results

### Baseline Data

Baseline data of patients in two groups are given in Table 1. As shown, there was no significantly different between these two groups in the baseline data including gender, age, BMI, fracture time, and fractured segment (*P *> 0.05), while the hospital stay time of patients in the PKP group was significantly longer than that in the Con group (*P *< 0.05).

**Table 1:** Baseline data in two groups.

Groups	Con group (*n* = 30)	PKP group (*n* = 30)	*t*/*χ*^2^	*P*
Male/female (*n*)	13/17	14/16	0.052	0.820
Age (year)	67.03 ± 14.17	67.07 ± 4.03	0.031	0.975
BMI (kg/m^2^)	24.78 ± 0.86	24.45 ± 0.58	1.736	0.088
Fracture time (day)	4.57 ± 1.01	4.67 ± 0.76	0.435	0.665
Hospital stay (day)	8.13 ± 0.86	12.97 ± 0.93	20.921	<0.001
Fractured segment (*n*)				
T11	3	4	0.636	0.888
T12	9	7		
L1	11	13		
L2	7	6		

*BMI, body mass index*.

### Pain Severity

**Figure 1:**
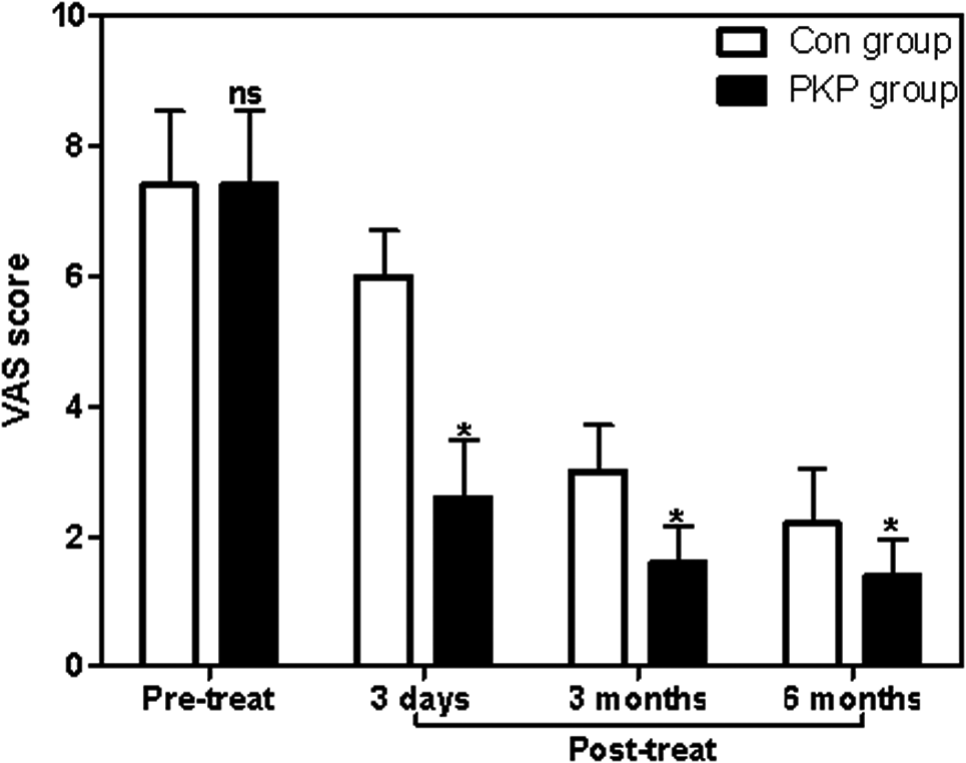
Comparison of VAS scores at different times between two groups. Note: Compared with the Con group, ^ns^*P* > 0.05 and *P < 0.05.

Before treatment, the VAS score of patients in the PKP group has no significant difference to patients in the Con group (*P *> 0.05). However, after different treatment methods, the VAS scores of patients in the PKP group were all significantly lower than those in the Con group at 3 days, 3 months, and 6 months post-treatment (*P *< 0.05) (Figure 1). At the same time, the scores of the four dimensions of the Dallas pain questionnaire scale in the PKP group were all significantly lower than those in the Con group at 6 months post-treatment (*P *< 0.05) (Table 2).

**Table 2:** Comparison of Dallas pain questionnaire at 6 months post-treatment between two groups.

Group	*n*	Daily life	Word and play	Anxiety and depression	Social interest
Con group	30	41.07 ± 3.62	42.57 ± 4.15	33.60 ± 5.13	33.6 ± 5.13
PKP group	30	26.73 ± 4.86	34.90 ± 2.87	16.27 ± 2.79	16.10 ± 3.46
*t*		12.963	8.324	17.387	15.494
*P*		<0.001	<0.001	<0.001	<0.001

### Recovery of Injured Vertebrae

**Figure 2:**
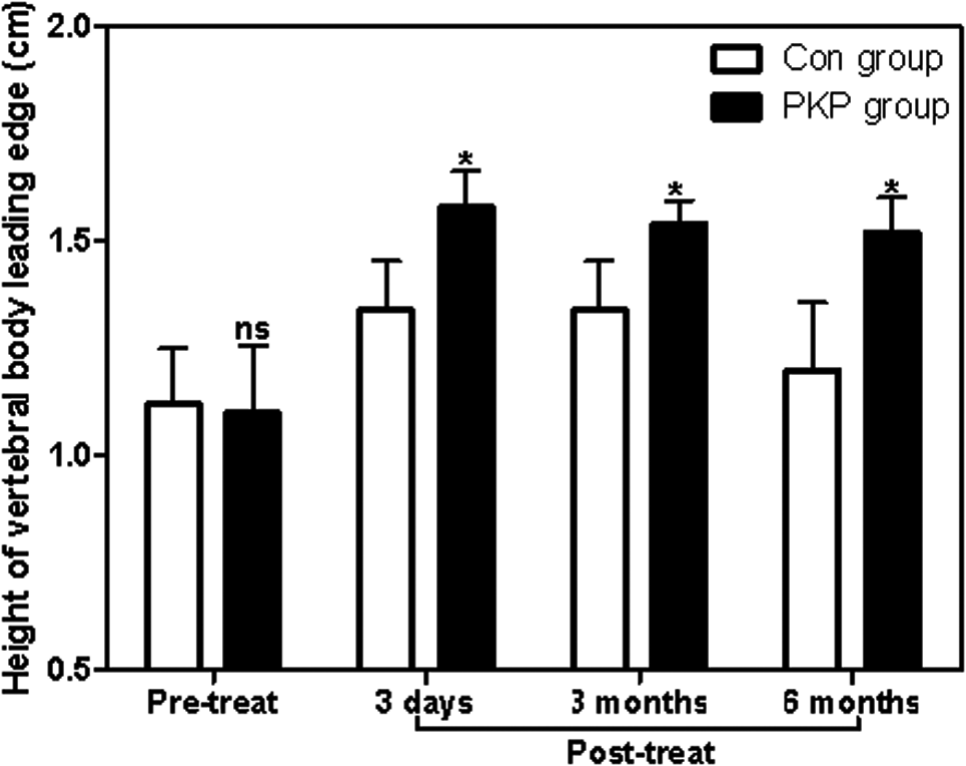
Comparison of the vertebral body leading edge height at different times between two groups. Note: Compared with the Con group, ^ns^*P* > 0.05 and *P < 0.05.

**Figure 3:**
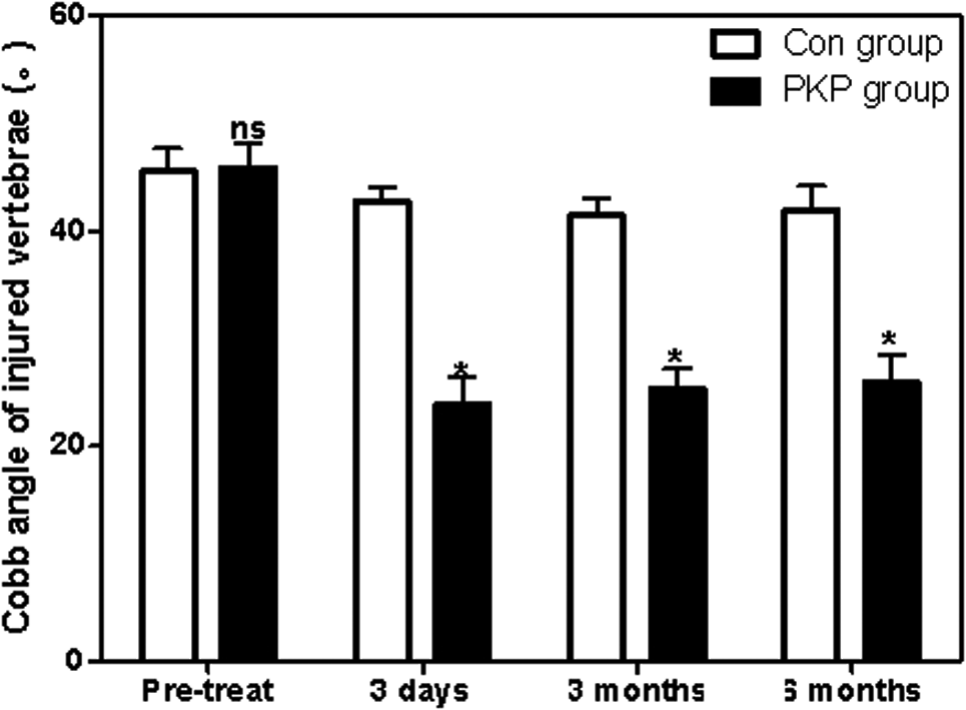
Comparison of the Cobb angle of injured vertebrae at different times between two groups. Note: Compared with the Con group, ^ns^*P* > 0.05 and *P < 0.05.

**Figure 4:**
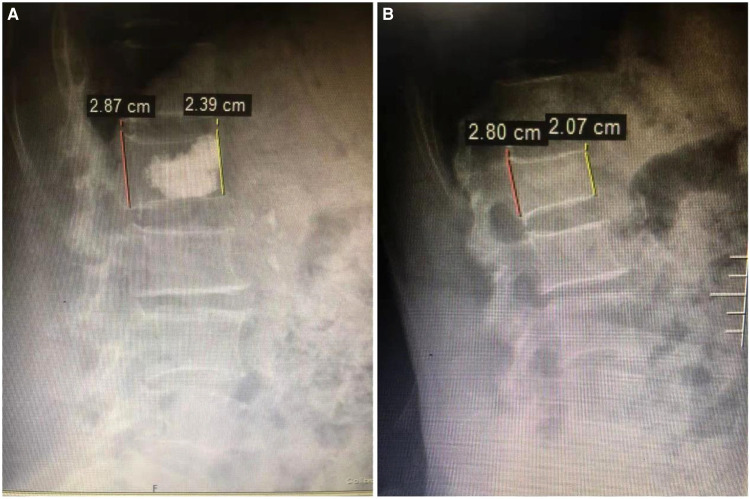
Changes in the height of the anterior vertebral body detected by X-ray before (**A**) and after (**B**) PKP treatment.

Before treatment, there was no significant difference in the anterior height between Con and PKP groups (1.21 ± 0.12 vs. 1.19 ± 0.11) (*P* > 0.05). However, after treatment with different methods, the heights of the anterior border of vertebral bodies in the PKP group were significantly higher than those in the Con group (1.35 ± 0.24 vs. 1.61 ± 0.23), (1.36 ± 0.23 vs. 1.58 ± 0.21), (1.30 ± 0.25 vs. 1.87 ± 0.22) (*P* < 0.05) 3 days, 3 months, and 6 months after treatment, respectively (Figure 2). Similarly, there was no significant difference in the Cobb angle of the injured vertebral body between the Con group and the PKP group (46.58 ± 2.71 vs. 46.71 ± 2.76) (*P* > 0.05). After treatment with different methods, the Cobb angles of the injured vertebra in the PKP group were significantly lower than those in the control group (*P* < 0.05) (45.15 ± 2.84 vs. 25.17 ± 3.66), (43.27 ± 2.56 vs 27.33 ± 3.74), (44.28 ± 2.19 vs. 28.41 ± 3.71), 3 days, 3 months, and 6 months after treatment, respectively (Figures 3, 4).

### Other Clinical Outcomes

At 6 months post-treatment, the score of SF-36 (PCS), SF-36 (MCS), and Barthel index of patients in the PKP group were all significantly lower than those in the Con group (*P *< 0.05), while there was no significant difference in the scores of MMSE between these two groups (*P *> 0.05) (Table 3).

**Table 3:** Comparison of other clinical outcomes at 6 months post-treatment between two groups.

Group	*n*	SF-36 (PCS)	SF-36 (MCS)	Barthel	MMSE
Con group	30	36.27 ± 3.79	58.17 ± 5.95	26.83 ± 6.06	88.00 ± 4.57
PKP group	30	30.60 ± 5.95	47.96 ± 4.89	18.97 ± 4.10	87.50 ± 6.06
*t*		4.400	7.252	5.888	0.361
*P*		<0.001	<0.001	<0.001	0.719

*SF-36, MOS item short-form health survey; PCS, standardized physical component; MCS, standardized mental component; MMSE, mini-mental state examination*.

## Discussion

Elderly OVCF is one of the most common complications of osteoporosis, and the pathological characteristics of elderly osteoporosis are closely related to systemic functional decline. Surveys have found that the mortality rate of elderly OVCF patients within 5 years is as high as 23%–34% (16). Therefore, it is urgent to find an effective therapeutic scheme in the clinic.

It is a specific and prominent symptom of systemic diseases. Conservative treatment and vertebral augmentation techniques are the most common treatment protocols for OVCF patients. Conservative treatment for OVCF such as bed rest, taking calcium and analgesic drugs, and wearing orthopedic braces are all require patients to stay in bed for a long time. However, long-term bed rest can cause dysfunction of the body, which is not only conducive to the recovery of bone volume but also easily accelerates the loss of bone mass, aggravates the pain, and causes muscle atrophy (17). Besides, for elderly OVCF patients, long-term bed rest treatment can also induce other diseases such as pneumonia and deep vein thrombosis, accelerate the deterioration of the disease, and even lead to death (18). Importantly, conservative treatment fails to quickly relieve pain symptoms in OVCF patients, which also aggravates the limitation of conservative treatment. Therefore, surgery is an effective option for OVCF. However, traditional open surgery is traumatic for patients with osteoporosis and prone to internal fixation loosening, which is only applicable to a few patients with symptoms of the spinal cord or nerve compression. In addition, since most OVCF injuries are nonviolent, generally, without symptoms of neurological damage or significant spinal instability, incision surgery and pedicle screw fixation are not required. In addition, due to the characteristics of elderly patients and osteoporosis, pedicle screws are prone to failure. Therefore, the efficacy of traditional open surgery is not significant enough in the clinical treatment of OVCF.

In this study, patients in the PKP group received PKP therapy, and we found that the score of VAS in OVCF patients in the PKP group is significantly lower than that in OVCF patients receiving conservative treatment at 3 days, 3 months, and 6 months post-treatment, while the scores of the four dimensions of the Dallas pain questionnaire scale in the PKP group were all significantly lower than those in OVCF patients receiving conservative treatment at 6 months post-treatment. These results suggested that PKP treatment relieves pain in OCVF patients faster than conservative treatment. Consistent with previous studies, rapid pain relief is the biggest advantage of PKP over conservative treatment (19). PKP percutaneous balloon vertebroplasty is a microinnovative technique for spine surgery developed on the basis of percutaneous vertebroplasty (PVP) (20). The main protocol for PKP treatment of OVCF is as follows: under the monitoring of imaging equipment, a balloon is inserted and inflated with minimally invasive techniques until the endplate is elevated, the height of the vertebral body is restored satisfactorily, and a cavity is formed in the vertebral body (21). Methyl methacrylate—bone cement—is injected into the vertebral body through the skin and pedicle to fill it, restore the height of the vertebral body, increase the strength of the diseased vertebral body, prevent further collapse and refracture of the vertebral body, correct the kyphosis deformity, relieve pain, and improve physical function so that patients can get out of bed early (22).

In the present, we also found that the Cobb angle of injured vertebrae of OVCF patients in the PKP group is significantly lower than those of OVCF patients receiving conservative treatment, while the heights of the vertebral body leading edge in the PKP group were all significantly lower than those in OVCF patients receiving conservative treatment at 3 days, 3 months, and 6 months post-treatment (23). Therefore, the above results indicated that injured vertebral bodies recovered faster in OVCF patients treated with PKP than those treated with conservative treatment. Furthermore, both pain and vertebral function recovery impact the quality of life and mental status of OVCF patients (24). Although the long-term improvement of pain and functional recovery in acute OVCF patients treated with PKP was not significantly different from conservative treatment in some previous studies, it should be noted that the quality of life and mental status of patients treated with PKP were better than those treated with conservative treatment, which was consistent with conservative treatment. PKP therapy is associated with rapid pain relief and restoration of vertebral function. Consistently, in this study, we found that the scores of SF-36 (PCS), SF-36 (MCS), and Barthel index of patients in the PKP group were all significantly lower than those in the Con group at 6 months post-treatment, which suggested that the quality of life and mental status of patients treated with PKP were better than those treated with conservative treatment. In addition, prolonged hospitalization due to preoperative MRI and bone mineral density testing resulted in a longer average hospitalization in the PKP group than that in the Con group in this study. Patients need to be informed of the operation before the operation to improve the patients’ informed degree of the operation.

## Conclusion

Compared with conservative treatment, PKP treatment of elderly patients with acute symptomatic OVCF provides rapid pain relief, restoration of damaged vertebral body height, correction of Cobb's angle, and improved quality of life. However, the high cost of treatment and the increased risk of postoperative refracture are the disadvantages of PKP treatment for OVCF patients.

## Data Availability

The original contributions presented in the study are included in the article/Supplementary Material; further inquiries can be directed to the corresponding author/s.
